# Behavioral Phenotypes and Comorbidity in 3q29 Deletion Syndrome: Results from the 3q29 Registry

**DOI:** 10.1007/s10803-023-06218-w

**Published:** 2024-01-12

**Authors:** Rebecca M. Pollak, Michael Mortillo, Melissa M. Murphy, Jennifer G. Mulle

**Affiliations:** 1 Department of Psychiatry, Center for Advanced Biotechnology and Medicine, Robert Wood Johnson Medical School, Rutgers University, 679 Hoes Ln W, Piscataway, NJ 08854 USA; 2 Rollins School of Public Health, Emory University, Atlanta, GA USA; 3 Department of Pediatrics, School of Medicine, Emory University, Atlanta, GA USA; 4 Department of Psychiatry, Robert Wood Johnson Medical School, Rutgers University, New Brunswick, NJ USA

**Keywords:** 3q29 deletion syndrome, CBCL, ABCL, Rare disorder, CNV

## Abstract

3q29 deletion syndrome (3q29del) is associated with a significantly increased risk for neurodevelopmental and neuropsychiatric disorders. However, the full spectrum of behavioral phenotypes associated with 3q29del is still evolving. Individuals with 3q29del (n = 96, 60.42% male) or their guardian completed the Achenbach Child or Adult Behavior Checklist (CBCL/ABCL) via the online 3q29 registry (3q29deletion.org). Typically developing controls (n = 57, 49.12% male) were ascertained as a comparison group. We analyzed mean performance on the CBCL/ABCL for individuals with 3q29del and controls across composite, DSM-keyed, and developmental scales; and the relationship between CBCL/ABCL performance and clinical and developmental phenotypes for individuals with 3q29del. Individuals with 3q29del showed significantly elevated behavioral and developmental impairment relative to controls across CBCL/ABCL domains. A substantial proportion of study participants with 3q29del scored in the Borderline or Clinical range for composite and DSM-keyed scales, indicating significant behavioral problems that may require clinical evaluation. We found that the preschool CBCL DSM-keyed autism spectrum problems scale is a potential screening tool for autism spectrum disorder (ASD) for individuals with 3q29del; CBCL/ABCL DSM-keyed scales were not accurate screeners for anxiety disorders or attention-deficit/hyperactivity disorder (ADHD) in our study sample. We identified a high degree of psychiatric comorbidity in individuals with 3q29del, with 60.42% (n = 58) of individuals with 3q29del scoring in the Borderline or Clinical range on two or more DSM-keyed CBCL/ABCL scales. Finally, we found that the degree of developmental delay in participants with 3q29del does not explain the increased behavioral problems observed on the CBCL/ABCL. The CBCL/ABCL can be used as screening tools in populations such as 3q29del, even in the presence of substantial psychiatric comorbidity. These results expand our understanding of the phenotypic spectrum of 3q29del and demonstrate an effective method for recruiting and phenotyping a large sample of individuals with a rare genetic disorder.

**Supplementary Information:**

The online version contains supplementary material available at 10.1007/s10803-023-06218-w.

## Introduction

3q29 deletion syndrome (3q29del) is a rare (1:30,000) (Kendall et al., 2017; Stefansson et al., 2014) genetic disorder associated with significant neurodevelopmental and psychiatric disability, including a greater than 40-fold increased risk for schizophrenia (SZ) (Kirov et al., 2012; Marshall et al., 2017; Mulle, 2015; Mulle et al., 2010; Szatkiewicz et al., 2014). Individuals with 3q29del are also at 19-fold increased risk for autism spectrum disorder (ASD) (Itsara et al., 2009; Pollak et al., 2019, 2022a; Sanders et al., 2015), as well as mild to moderate intellectual disability and anxiety disorders (Ballif et al., 2008; Cox & Butler, 2015; Girirajan et al., 2012; Glassford et al., 2016; Klaiman, 2022; Sanchez Russo et al., 2021; Willatt et al., 2005). Recent work by our team has also identified increased rates of attention deficit/hyperactivity disorder (ADHD), executive function deficits, graphomotor weakness, and adaptive behavior deficits (Klaiman, 2022; Pollak, 2023a, 2023b; Sanchez Russo et al., 2021). 3q29del is defined by the presence of the 3q29 deletion, a rare (1:30,000) (Kendall et al., 2017; Stefansson et al., 2014), typically de novo 1.6 Mb deletion near the telomere of the long arm of chromosome 3 (hg19, chr3:195,725,000–197,350,000) (Ballif et al., 2008; Glassford et al., 2016; Willatt et al., 2005). The phenotypic spectrum of 3q29del shares elements with other rare genetic disorders, including 22q11.2 deletion syndrome and 16p11.2 deletion and duplication syndromes (D'Angelo, et al., 2016; Hanson, et al., 2015; McDonald-McGinn, et al., 2005; Schneider, et al., 2014; Steinman, et al., 2016). The heterogeneity of these disorders, and the repeated observation that one variant can increase risk for multiple neurodevelopmental and psychiatric conditions, has contributed to new hypotheses about the potential for shared etiology across phenotypes. To fully explore these potential relationships, it is critical to rigorously document the range of neurodevelopmental and psychiatric morbidity associated with rare genetic variants, including the 3q29 deletion.

There are significant challenges to uncovering the full phenotypic spectrum of a rare genetic disorder like 3q29del. Much of our current understanding of rare disorders stems from case reports; while useful, case reports often do not collect systematic or comprehensive phenotypic data, and the number of affected individuals described in any one case report tends to be small. Additionally, the measures and data collection instruments used are rarely harmonized across case studies, which can lead to bias when aggregating data. Most critically, the low population frequency of rare genetic disorders represents a significant barrier that can prevent any individual researcher from amassing a large enough sample size for meaningful phenotyping studies. To address these obstacles, in 2014 our team created an online registry for individuals with 3q29del (Glassford et al., 2016). In the registry, affected individuals and their caregivers have the opportunity to fill out standardized self-report phenotyping instruments. This registry has been particularly useful to document our emerging understanding of the 3q29del phenotype (Glassford et al., 2016; Pollak, 2023c; Pollak et al., 2019; Wawrzonek et al., 2022). Registries for other rare genetic disorders have been met with similar success (Boulanger et al., 2020; Jonker et al., 2022; Zilber et al., 2023).

In the present study, we further define the phenotypic spectrum of 3q29del, leveraging the power of the online 3q29del registry. We evaluated data from the Achenbach Child and Adult Behavior Checklists from the largest cohort of individuals with 3q29del to date and found that the 3q29 deletion is associated with elevated emotional, behavioral, and social problems relative to a sample of typically developing controls. Individuals with 3q29del also demonstrate extensive comorbidity across multiple functional domains, supporting data from case reports and in-person phenotyping studies and highlighting the importance of considering comorbid diagnoses when evaluating or treating an individual with 3q29del. Developing a more detailed understanding of the phenotypic spectrum of 3q29del using standardized instruments will help to guide patients, families, and clinicians in proper management of the syndrome, and may provide insight into potential areas for early intervention to improve long-term outcomes.

## Methods

### Study Participants

Individuals with 3q29del were recruited from the online 3q29 registry (3q29deletion.org), a web-based platform that allows for online recruitment, informed consent, and data collection (Glassford et al., 2016). Typically developing controls were recruited to participate in the 3q29 registry as previously described (Pollak et al., 2019). 96 individuals with 3q29del (60.42% male) and 57 typically developing controls (49.12% male) were included in the present study. Study participants with 3q29del ranged in age from 1.80 to 66.60 years (mean = 10.92 ± 8.33 years), typically developing controls ranged in age from 1.67 to 41.60 years (mean = 10.02 ± 6.95 years). See Table 1 for a description of the study sample. 29 of the total cohort of 96 individuals with 3q29del also participated in an in-person deep phenotyping study conducted by our team as previously described (Murphy et al., 2018; Sanchez Russo et al., 2021). This study was approved by Emory University’s Institutional Review Board (IRB00064133) and Rutgers University’s Institutional Review Board (PRO2021001360).

**Table 1:** Demographic information for study participants with 3q29 deletion syndrome (n = 96) and typically developing controls (n = 57)

	3q29del		Control		p value
	Mean ± SD	Range	Mean ± SD	Range	
Age (years)	10.92 ± 8.33	1.80–66.60	10.02 ± 6.95	1.67–41.60	0.473

	N	%	N	%	
Sex					0.182
Male	58	60.42	28	49.12	
Female	38	39.58	29	50.88	
Race					0.001
White	82	85.42	38	66.67	
Black	1	1.04	9	15.79	
Other	13	13.54	8	14.04	
Unknown/not reported	0	0.00	2	3.51	
Ethnicity					0.41
Not Hispanic/Latino	74	77.08	49	85.96	
Hispanic/Latino	9	9.38	4	7.02	
Unknown/not reported	13	13.54	4	7.02	
Questionnaire form					0.966
Preschool CBCL	32	33.33	18	31.58	
School age CBCL	56	58.33	35	61.40	
ABCL	8	8.33	4	7.02	

### Measures

Upon joining the 3q29 registry, the individual with 3q29del or their caregiver completed a custom medical and demographic questionnaire (Glassford et al., 2016). This questionnaire collected demographic information including sex, age, race, and ethnicity of the individual with 3q29del. It also collected a detailed medical history, including any professional diagnoses of neurodevelopmental or psychiatric disorders and developmental milestones.

The Achenbach Child Behavior Checklist (CBCL) and Adult Behavior Checklist (ABCL) are 100-, 113-, or 126-item (preschool CBCL, school age CBCL, ABCL) 3-point Likert-scaled questionnaires (Achenbach & Rescorla, 2000; Achenbach et al., 2001, 2003). The preschool CBCL is for children ages 1.5–5 years, the school age CBCL is for children ages 6–18 years, and the ABCL is for adults over age 18. The preschool CBCL also includes a Language Development Survey (LDS) that measures Phrase Development and Vocabulary Development. The Phrase Development scale is for children ages 24–35 months, and the Vocabulary Development scale is for children ages 18–35 months. The school age CBCL includes a Competence section that measures the child’s involvement and skill in Activities, Social life, School, and a composite Total Competence scale. The ABCL includes an Adaptive Functioning section that measures the individual’s relationships with Friends and with their Spouse/Partner; none of our respondents reported living with a Spouse/Partner over the past 6 months, so this scale was excluded from the Adaptive Functioning section. Each questionnaire includes sections targeting different age-appropriate behavioral and developmental problems, as well as expert-derived scales keyed to specific Diagnostic and Statistical Manual (DSM) diagnoses. Because the DSM-keyed scales are not diagnostic measures, the scales indicate disorder-relevant problems (for example, the Autism spectrum problems scale or the Attention-deficit/hyperactivity problems scale); high scores on these scales do not necessarily indicate a diagnosis of the disorder. Each questionnaire also includes composite scores that measure overall internalizing and externalizing behaviors. The CBCL/ABCL scales used in the present analysis are depicted in Fig. S1. Scores on the CBCL and ABCL are converted to T-scores that are normed by sex and age. There are defined cutoffs to categorize the severity of problems on each scale as Normal, Borderline, or Clinical; the specific numeric cutoffs vary between scale types (Table S1).

A subset of 29 individuals with 3q29del participated in an in-person deep phenotyping study in addition to completing the online CBCL or ABCL (Murphy et al., 2018; Sanchez Russo et al., 2021). Subjects were assessed for neurodevelopmental and psychiatric phenotypes using gold-standard diagnostic assessments administered by expert clinicians (Murphy et al., 2018; Sanchez Russo et al., 2021). In the present study, diagnoses of anxiety disorders and ADHD (Murphy et al., 2018; Sanchez Russo et al., 2021) from the in-person deep phenotyping study were used to test if the CBCL and ABCL DSM-keyed scales are accurate screening tools for those disorders in individuals with 3q29del.

### Analysis

All analyses were performed in R version 4.0.4 (R Core Team, 2008). Data were securely downloaded from the online 3q29 registry and de-identified for analysis. Data were pooled across the preschool CBCL, school age CBCL, and ABCL for the Total Problems, Internalizing, Externalizing, DSM depressive problems, DSM anxiety problems, and DSM Attention Deficit/Hyperactivity (AD/H) scales. The preschool CBCL was analyzed independently for preschool CBCL-specific scales: DSM autism spectrum problems, DSM oppositional defiant problems, Vocabulary Development, and Phrase Development scales. The school age CBCL was analyzed independently for school age CBCL-specific scales: DSM somatic problems, DSM oppositional defiant problems, DSM conduct problems, Activities, Social, School, and Competence scales. The ABCL was analyzed independently for ABCL-specific scales: DSM somatic problems, DSM avoidant personality problems, DSM antisocial personality problems, and Friends scales. Age at walking was binned as normal (≤ 18 months), delayed (19–24 months), and extremely delayed (≥ 24 months), as previously described (Pollak et al., 2019). Statistical analysis was performed using Fisher’s exact tests, Student’s t tests, and simple linear models implemented using the stats R package (R Core Team, 2008). All models adjusted for age and sex. Receiver operating characteristic (ROC) curves were constructed using the ROCit R package (Khan et al., 2020). Data visualization was performed using the plotly R package (Sievert, et al., 2017).

## Results

### Individuals with 3q29del Show Significant Behavioral Impairment on the CBCL and ABCL Relative to Typically Developing Controls

**Fig. 1:**
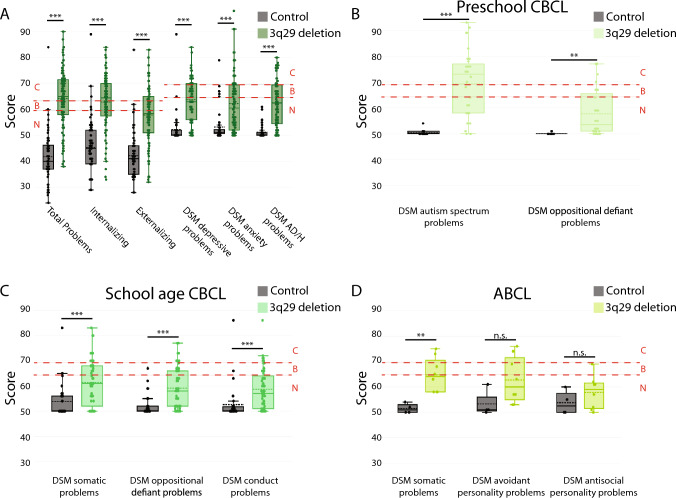
Distribution of scores on the **A** shared CBCL/ABCL composite and DSM-keyed scales (n = 96 3q29del, 57 control), **B** DSM-keyed scales unique to the preschool CBCL (n = 32 3q29del, 18 control), **C** DSM-keyed scales unique to the school age CBCL (n = 56 3q29del, 35 control), and **D** DSM-keyed scales unique to the ABCL (n = 8 3q29del, 4 control) for study participants with 3q29del and controls. Dashed red lines denote the cutoffs for the Borderline and Clinical score classifications. *N* normal, *B* borderline, *C* clinical, *AD/H* attention-deficit/hyperactivity, *n.s.* not significant; **p < 0.01; ***p < 0.001

On average, participants with 3q29del scored significantly higher than typically developing controls on the composite and DSM-keyed scales of the CBCL and ABCL (Fig. 1). Of the composite scales, the largest difference between participants with 3q29del and controls was on the Total Problems scale (3q29del mean = 64.25 ± 11.02, control mean = 41.75 ± 9.81, p < 2E−16; Fig. 1A), and the smallest difference was on the Externalizing scale (3q29del mean = 57.85 ± 11.66, control mean = 42.18 ± 9.36, p = 7.37E−15; Fig. 1A), with an intermediate difference on the Internalizing scale (3q29del mean = 62.84 ± 10.67, control mean = 45.33 ± 10.98, p < 2E−16; Fig. 1A). Notably, the mean score for participants with 3q29del was in the Clinical range for the Total Problems scale and the Borderline range for the Internalizing scale, indicating significantly elevated behavioral problems that may require clinical evaluation. The difference in scores between participants with 3q29del and controls was roughly equivalent across the three DSM-keyed scales that were included on all forms of the CBCL and ABCL; participants with 3q29del scored significantly higher than controls on all scales (DSM depressive problems 3q29del mean = 63.77 ± 8.56, control mean = 52.28 ± 5.69, p = 8.39E−16; DSM anxiety problems 3q29del mean = 62.29 ± 10.97, control mean = 53.21 ± 5.82, p = 3.66E−8; DSM AD/H problems 3q29del mean = 62.61 ± 9.20, control mean = 51.30 ± 2.63, p = 1.51E−15; Fig. 1A). While the mean scores for participants with 3q29del on the common DSM-keyed scales were in the Normal range, between 38.54 and 48.96% of participants with 3q29del scored in the Borderline or Clinical range, compared to 0.00% to 10.53% of controls, highlighting the increased psychiatric liability in this population.

To continue exploring the neurodevelopmental and psychiatric liability associated with 3q29del, we analyzed the form-specific DSM-keyed scales and developmental measures. For participants that completed the preschool CBCL (n = 32 3q29del, 18 control), individuals with 3q29del scored significantly higher than control participants on both DSM-keyed subscales (DSM autism spectrum problems 3q29del mean = 69.38 ± 12.90, control mean = 50.44 ± 0.98, p = 3.80E−6; DSM oppositional defiant problems 3q29del mean = 57.72 ± 8.63, control mean = 50.11 ± 0.32, p = 0.002; Fig. 1B). The mean score on the DSM autism spectrum problems scale for participants with 3q29del is in the Clinical range, consistent with prior work by our team and others demonstrating a significantly increased risk for ASD in individuals with 3q29del (Pollak et al., 2019, 2022a). We also performed an exploratory analysis of the LDS portion of the preschool CBCL. We found that significantly more participants with 3q29del were classified as Delayed for both the Phrase Development (n = 5 3q29del Delayed, 0 control Delayed, p = 0.015; Fig. S3A) and Vocabulary Development (n = 6 3q29del Delayed, 0 control Delayed, p = 0.0001; Fig. S3B) scales. These data are aligned with the increased rate of speech delay reported in individuals with 3q29del (Ballif et al., 2008; Biamino, et al., 2016; Città et al., 2013; Cox & Butler, 2015; Girirajan et al., 2012; Glassford et al., 2016; Quintero-Rivera et al., 2010; Sagar et al., 2013; Willatt et al., 2005). For participants that completed the school age CBCL (n = 56 3q29del, 35 control), individuals with 3q29del scored significantly higher than controls on all DSM-keyed scales (DSM somatic problems 3q29del mean = 61.34 ± 9.21, control mean = 53.77 ± 6.59, p = 6.25E−5; DSM oppositional defiant problems 3q29del mean = 59.11 ± 8.02, control mean = 52.03 ± 3.92, p = 3.94E−6; DSM conduct problems 3q29del mean = 58.70 ± 8.11, control mean = 52.63 ± 6.83, p = 2.37E−4; Fig. 1C).

We also analyzed the Competence portion of the school age CBCL as a measure of gross adaptive function in our study participants. Prior work by our team has demonstrated substantial deficits in adaptive ability in individuals with 3q29del (Klaiman, 2022; Pollak, 2023b; Sanchez Russo et al., 2021). It is important to note that on the Competence section, lower scores correspond to *worse* function in a given domain, opposite of the scoring for the rest of the school age CBCL. We found that participants with 3q29del had significantly lower scores compared to controls across all Competence section measures, indicating poorer function in all domains. The largest difference in scores was in the composite Competence scale (3q29del mean = 28.83 ± 7.62, control mean = 51.89 ± 9.05, p < 2E−16; Fig. S3C). Of the three Competence section sub-scales, individuals with 3q29del performed worst on the School scale (3q29del mean = 30.04 ± 8.04, control mean = 51.26 ± 6.24, p < 2E−16; Fig. S3C) and best on the Activities scale (3q29del mean = 36.23 ± 8.16, control mean = 49.14 ± 7.78, p = 1.3E−10; Fig. S3C), with an intermediate performance on the Social scale (3q29del mean = 34.54 ± 8.18, control mean = 51.09 ± 8.92, p = 3.38E−14; Fig. S3C). The average score for individuals with 3q29del on the composite Competence scale and the School scale was in the Clinical range, indicating substantial impairment; the average score on the Social scale was in the Borderline range. For participants that completed the ABCL (n = 8 3q29del, 4 control), individuals with 3q29del scored significantly higher than controls on the DSM somatic problems scale (3q29del mean = 64.75 ± 6.80, control mean = 51.50 ± 1.91, p = 0.004; Fig. 1D). There was no statistically significant difference in scores for the DSM avoidant personality problems scale (3q29del mean = 62.75 ± 9.24, control mean = 53.25 ± 5.19, p = 0.169; Fig. 1D) or the DSM antisocial personality problems scale (3q29del mean = 57.88 ± 6.60, control mean = 53.75 ± 4.79, p = 0.178; Fig. 1D), though the lack of statistical significance may be due to the small sample size for the ABCL. We also analyzed the Adaptive Function section of the ABCL, which is scored similarly to the Competence section of the school age CBCL, where lower scores correspond to *worse* function. Individuals with 3q29del scored significantly lower than controls on the Friends scale (3q29del mean = 38.88 ± 7.94, control mean = 51.25 ± 7.80, p = 0.046; Fig. S3D), indicating poorer function in this domain relative to controls. Taken together, these data highlight the high degree of neurodevelopmental and psychiatric liability associated with the 3q29 deletion across the lifespan and reinforce other published studies with evidence for neurodevelopmental and psychiatric phenotypes associated with 3q29del (Ballif et al., 2008; Cox & Butler, 2015; Klaiman, 2022; Murphy et al., 2020; Pollak, 2023a, 2023b; Pollak et al., 2019, 2022a; Sanchez Russo et al., 2021; Willatt et al., 2005).

### DSM-Keyed CBCL and ABCL Scales are not Accurate Screening Tools for all Phenotypes in Individuals with 3q29del

**Fig. 2:**
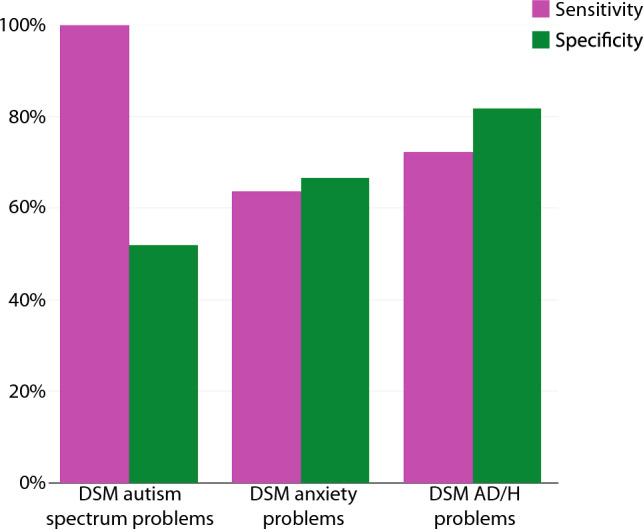
Sensitivity and specificity metrics for the preschool CBCL autism spectrum problems scale and the CBCL/ABCL anxiety problems and attention-deficit/hyperactivity problems scales. *AD/H* attention-deficit/hyperactivity

3q29del is a rare disorder; as such, it can be difficult to amass a substantial cohort for meaningful phenotyping studies. Deploying standardized online and remote measures to screen study participants for specific phenotypes of interest can be a powerful way to identify sub-populations that would benefit from gold-standard assessments or recruitment for specialized study design. We tested whether the CBCL and ABCL DSM-keyed scales could accurately discriminate between participants with 3q29del with and without ASD, anxiety disorder, and ADHD. Work by our team and others has demonstrated that the 3q29 deletion confers elevated risk for all three of these diagnoses (Glassford et al., 2016; Itsara et al., 2009; Klaiman, 2022; Pollak et al., 2019, 2022a; Sanchez Russo et al., 2021; Sanders et al., 2015); identifying effective screening tools could help to prioritize individuals for gold-standard assessments and early interventions. To test the effectiveness of the CBCL as a screener for ASD, we used the preschool CBCL DSM autism spectrum problems scale, using participant-reported clinical diagnosis of ASD as “ground truth”. We used participant-reported diagnosis of ASD for this analysis because there was not sufficient overlap between study participants that completed the preschool CBCL and those that participated in a previously published deep phenotyping study by our team (Klaiman, 2022; Sanchez Russo et al., 2021). We used the Borderline cutoff for the preschool CBCL DSM autism spectrum problems scale for this analysis. We found that the preschool CBCL DSM autism spectrum problems scale had a sensitivity rate of 100% in our study population, indicating that all participants reporting an ASD diagnosis screened positive on the scale (n = 7), and a specificity of 52%, indicating that 48% of individuals that screened positive did not report a diagnosis of ASD (n = 12) (Figs. 2, S2A). To assess the classification accuracy for diagnoses of anxiety disorder and ADHD, we used the DSM anxiety problems and DSM AD/H problems scales that are included on all forms of the CBCL and ABCL. We used the Borderline cutoff of the CBCL/ABCL DSM anxiety problems and AD/H problems scales for this analysis. We tested these scales against previously published gold-standard diagnoses from the cohort of 29 individuals with 3q29del that participated in our deep phenotyping study (Klaiman, 2022; Sanchez Russo et al., 2021). We found that the DSM anxiety problems scale had a sensitivity rate of 63.6%, indicating that 36.4% of individuals with an anxiety disorder diagnosis did *not* screen positive on the scale (n = 4), and a specificity of 66.7%, indicating that 33.3% of individuals that screened positive did not have an anxiety disorder diagnosis (n = 6) (Klaiman, 2022; Sanchez Russo et al., 2021) (Figs. 2, S2B). We found that the DSM AD/H problems scale had a sensitivity rate of 72.2%, indicating that 27.8% of individuals with an ADHD diagnosis did *not* screen positive on the scale (n = 5), and a specificity rate of 81.8%, indicating that 18.2% of individuals that screened positive did not have an ADHD diagnosis (n = 2) (Klaiman, 2022; Sanchez Russo et al., 2021) (Figs. 2, S2C). Together, these data show that the preschool CBCL autism spectrum problems scale is an accurate screening tool for ASD in individuals with 3q29del, but the CBCL and ABCL are not accurate screeners for anxiety disorders or ADHD in this population.

### Individuals with 3q29del Show a High Degree of Comorbidity on the CBCL and ABCL

**Fig. 3:**
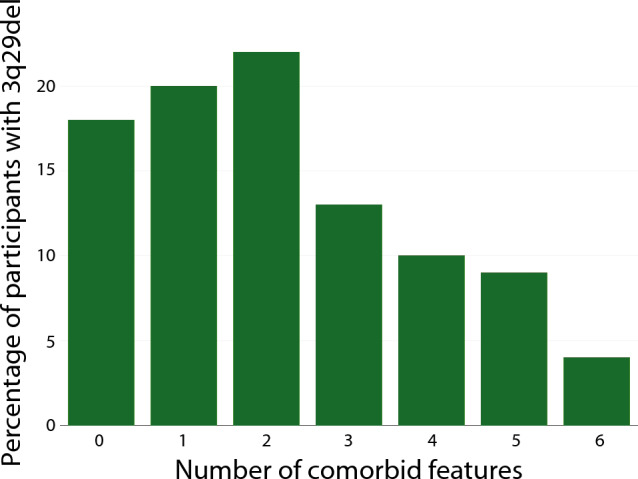
Distribution of the number of comorbid features, defined as scores on DSM-keyed scales in the Borderline or Clinical range, for study participants with 3q29del

Previous work by our team has highlighted the substantial presence of neurodevelopmental and psychiatric *comorbidity* associated with the 3q29 deletion (Klaiman, 2022; Pollak, 2023b; Sanchez Russo et al., 2021); comorbidity is also a feature of other rare genetic disorders including 22q11.2 deletion syndrome and 16p11.2 deletion and duplication syndromes (D'Angelo, et al., 2016; Hanson, et al., 2015; McDonald-McGinn, et al., 2005; Schneider, et al., 2014; Steinman, et al., 2016). We sought to determine whether the CBCL and ABCL also capture this comorbidity in our sample of individuals with 3q29del. To test this, we used the DSM-keyed scales from all forms of the CBCL and ABCL and considered a score in the Borderline or Clinical range to be counted as a neurodevelopmental or psychiatric feature. We found that 60.42% (n = 58) of study participants with 3q29del scored in the Borderline or Clinical range on two or more DSM-keyed scales (Fig. 3). Only 18.75% (n = 18) of participants with 3q29del did not score in the Borderline or Clinical range on any scale, and 20.83% (n = 20) of participants with 3q29del scored in the Borderline or Clinical range on one scale (Fig. 3). Together, these data support prior findings of the high degree of comorbidity in individuals with 3q29del and suggest that this neurodevelopmental and psychiatric comorbidity may be a hallmark feature of the syndrome.

### Behavior Deficits on the CBCL and ABCL in Individuals with 3q29del are not Sex-Dependent

Prior work by our team has identified sex-dependent differences associated with the 3q29 deletion in both human patients and our mouse model (Pollak, 2023a; Pollak et al., 2019, 2022b; Rutkowski, 2019). To test whether there are significant sex-dependent differences in performance on the CBCL and ABCL, we conducted a sex-stratified analysis of study participants with 3q29del. There were no sex-dependent differences in any composite or DSM-keyed scale (Fig. S4). Together, these data show that there are no substantial sex-dependent differences for individuals with 3q29del in general behavioral problems as measured by the CBCL and ABCL.

### Developmental Delay Does not Explain Increased Behavior Problems in Individuals with 3q29del

**Fig. 4:**
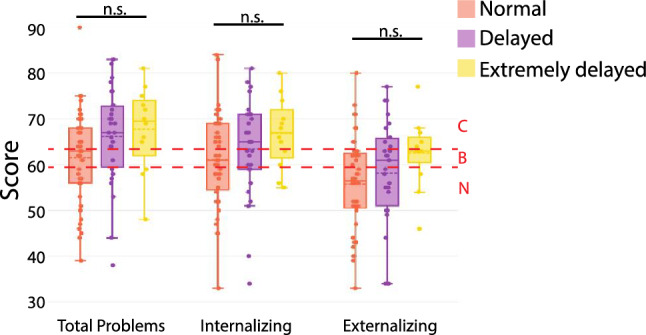
Distribution of scores on the CBCL/ABCL composite scales stratified by age at walking for study participants with 3q29del (n = 96). *n.s.* not significant

Due to the high rate of developmental delay and ID (DD/ID) associated with the 3q29 deletion (Ballif et al., 2008; Biamino, et al., 2016; Città et al., 2013; Cox & Butler, 2015; Girirajan et al., 2012; Glassford et al., 2016; Klaiman, 2022; Quintero-Rivera et al., 2010; Sagar et al., 2013; Sanchez Russo et al., 2021; Willatt et al., 2005), we sought to test whether the presence of DD/ID moderates the elevated scores on the CBCL and ABCL composite scales in participants with 3q29del. For this analysis we used parent-reported age at walking, which has been shown to be an accurate proxy for DD/ID (Bishop et al., 2016). Age at walking was not a strong predictor of CBCL and ABCL composite scores (Fig. 4). For the Total Problems scale, participants with 3q29del that were in the Normal age at walking category scored on average in the Borderline range, while participants in the Delayed and Extremely Delayed categories scored in the Clinical range (Normal mean = 61.55 ± 10.27, Delayed mean = 66.19 ± 10.71, Extremely Delayed mean = 67.75 ± 9.24, p = 0.089; Fig. 4). For the Internalizing scale, participants with 3q29del in the Normal and Delayed categories scored in the Borderline range, and participants in the Extremely Delayed category scored in the Clinical range (Normal mean = 61.10 ± 10.36, Delayed mean = 63.44 ± 10.96, Extremely Delayed mean = 66.83 ± 7.67, p = 0.206; Fig. 4). For the Externalizing scale, participants with 3q29del in the Normal and Delayed range scored in the Normal range, and participants in the Extremely Delayed category scored in the Borderline range (Normal mean = 55.80 ± 10.50, Delayed mean = 58.19 ± 11.89, Extremely Delayed mean = 62.58 ± 7.60, p = 0.159; Fig. 4). We also analyzed the relationship between age at walking and the degree of comorbidity for study participants with 3q29del. There was no relationship between the number of comorbid diagnoses and age at walking category (Normal mean = 1.90 ± 1.63, Delayed mean = 2.22 ± 1.63, Extremely Delayed mean = 2.92 ± 1.88, p = 0.184). Together, these data suggest that while there is a minor effect of age at walking, DD/ID is not the main driver of elevated scores on the CBCL and ABCL, and that the increased behavioral problems in individuals with 3q29del are a feature of the syndrome independent of DD/ID.

## Discussion

The present study uses the largest known cohort of individuals with 3q29del and typically developing controls to explore behavioral and developmental phenotypes associated with the 3q29 deletion. We found that individuals with 3q29del have significantly elevated behavioral and developmental challenges relative to typically developing controls as measured by the CBCL and ABCL. While the mean scores for individuals with 3q29del were elevated across scales, individual scores were highly variable, with some participants scoring in the Normal range, highlighting the phenotypic heterogeneity associated with the 3q29 deletion. We also report that the preschool CBCL DSM-keyed autism spectrum problems scale can discriminate between individuals with 3q29del and a diagnosis of ASD versus individuals with 3q29del and no diagnosis; however, the CBCL and ABCL DSM-keyed scales were not accurate screening tools for anxiety disorders or ADHD in this population. Finally, we found a high rate of comorbidity among participants with 3q29del, with a majority of individuals scoring in the Borderline or Clinical range on two or more DSM-keyed scales. This is consistent with prior work by our team identifying neurodevelopmental and psychiatric comorbidity as a feature of 3q29del (Klaiman, 2022; Pollak, 2023b; Sanchez Russo et al., 2021).

The study of rare genetic disorders like 3q29del has yielded important insights into the mechanistic underpinnings of common complex disorders like SZ and ASD (Gokhale, et al., 2019; Li et al., 2021; Marshall et al., 2017; Purcell, et al., 2023; Rubeis et al., 2014; Sanders et al., 2015; Satterstrom et al., 2020; Sefik et al., 2021). However, the insights gained from rare genetic disorders are limited by our ability to describe their complete phenotypic spectrum. The simple fact that these are rare disorders makes it inherently difficult to amass a sample size large enough for any substantial phenotyping effort. Here, we show that online participant recruitment and data collection represent an effective and robust way to conduct a phenotyping study of a rare disorder. By deploying standardized assessments through a confidential online patient registry, our team was able to measure behavioral vulnerabilities in a large cohort of individuals with 3q29del in an efficient and cost-effective manner. Further, we show that the preschool CBCL DSM-keyed autism spectrum problems scale is an accurate screening tool for ASD in individuals with 3q29del, with a sensitivity rate of 100% and a specificity rate of 52%. This 12-question subscale of the preschool CBCL could be deployed to prioritize individuals with 3q29del for gold-standard ASD evaluation; in the present study, all individuals that reported a diagnosis of ASD screened positive, and 48% of those not reporting a diagnosis screened negative. Using a screening tool like the preschool CBCL in this population would increase the likelihood that individuals that need an ASD evaluation would receive one, while simultaneously reducing the substantial financial and time burden associated with performing gold-standard ASD diagnostic assessments in individuals that do not require them. We also found that the CBCL and ABCL DSM-keyed scales do *not* accurately screen for anxiety disorders or ADHD in individuals with 3q29del, emphasizing the need for gold-standard diagnostic evaluations for those phenotypes for all individuals with 3q29del. Phenotypes such as anxiety disorders and ADHD may present differently in individuals with 3q29del as compared to idiopathic cases, resulting in the CBCL/ABCL misclassifying a significant fraction of individuals. The results of this study highlight the value of registry-based recruitment and phenotyping tools for rare disorders such as 3q29del and suggest that these methods may also benefit the study of other genetically susceptible populations.

Our team recently performed a deep phenotyping study of 32 individuals with 3q29del, where we identified substantial neurodevelopmental and psychiatric comorbidity associated with the 3q29 deletion (Klaiman, 2022; Murphy et al., 2018; Pollak, 2023b; Sanchez Russo et al., 2021); on average, study participants qualified for 3 separate diagnoses (range 0–5) (Pollak, 2023b). Case reports of individuals with 3q29del also typically report multiple neurodevelopmental and psychiatric diagnoses (Ballif et al., 2008; Biamino, et al., 2016; Città et al., 2013; Cox & Butler, 2015; Quintero-Rivera et al., 2010; Sagar et al., 2013; Willatt et al., 2005). In the present study, we found that a majority of participants with 3q29del scored in the Borderline or Clinical range on two or more DSM-keyed scales of the CBCL and ABCL, consistent with these prior findings. Further, this suggests that the cohort of individuals that participated in our deep phenotyping study was representative of the population of individuals with 3q29del at large, highlighting the role of comorbidity in this disorder and suggesting that this high degree of neurodevelopmental and psychiatric comorbidity is a hallmark feature of 3q29del. This comorbidity is an important consideration when thinking about evaluation, treatment strategies, and outcomes for an individual with 3q29del. Rather than treating diagnoses in silos with different specialist clinicians, individuals with 3q29del would likely benefit from an integrated treatment approach, where they receive care from a specialist team that communicates across disciplines to tailor interventions to each individual’s unique combination of strengths and vulnerabilities (Chawner et al., 2021; Ogundele & Morton, 2022). Diagnostic evaluations should also be performed by an integrated team; because one diagnosis may mask features of another, communication between medical professionals is critical to ensure that individuals with 3q29del receive complete and accurate diagnostic evaluations for neurodevelopmental and psychiatric disorders. Improved evaluation and treatment strategies through the use of an integrated team approach will enhance the accuracy of diagnosis and more specifically tailor interventions to the individual with 3q29del, likely improving long-term outcomes and quality of life.

In addition to emphasizing the need for integrated treatment of individuals with 3q29del, this study also highlights an important gap in evaluation and treatment. We found that the CBCL/ABCL DSM-keyed scales were not accurate screeners for anxiety disorders or ADHD; not all study participants that scored in the Borderline or Clinical range on these scales reported a diagnosis of the respective disorder. Additionally, some study participants scored in the Normal range on the CBCL/ABCL DSM-keyed scales even though they qualify for a diagnosis of anxiety disorders and/or ADHD. This discrepancy is likely due to two main sources. First, the DSM-keyed scales are not diagnostic proxies for these disorders; thus, individuals may score high on a given screener scale while not meeting the explicit diagnostic criteria for a disorder. The second, more damaging, possibility is that low CBCL/ABCL screener scores may not accurately capture the true degree of disability in some participants. We found that the CBCL/ABCL DSM-keyed scales correctly classified 63–72% of study participants, indicating that 28–36% of individuals with 3q29del may be inaccurately classified by these screening tools. These data suggest that these phenotypes may have an atypical presentation in some individuals with 3q29del, possibly due to the neurodevelopmental and psychiatric comorbidity in this population. Together, these data highlight the importance of improving healthcare delivery to evaluate and manage the neurodevelopmental and psychiatric manifestations associated with 3q29del. Gold-standard diagnostic evaluations should be standard of care for 3q29del; this will enable a higher percentage of individuals with 3q29del to access early intervention and other treatment strategies that will maximize outcomes for affected individuals and their families.

While this study is an important addition to the literature surrounding 3q29del, it is not without limitations. First, we were unable to assess the role of race and ethnicity in the present analysis, as our current sample is overwhelmingly white and non-Hispanic. Future recruitment efforts will aim to increase the number of study participants from under-represented minority groups. Second, the measures used in the present study are parent-report and may be subject to bias. However, the relatively high concordance between parent-reported diagnoses and screening scale scores, as well as previous work by our group (Pollak et al., 2019), suggests that these data are reliable. Additionally, individuals with 3q29del are typically diagnosed as children due to early-onset phenotypes such as congenital heart defects and developmental delays; therefore, our study population skews young (average age = 10.92 ± 8.33 years) and we were underpowered for some analyses, particularly the ABCL-specific scales. Future studies are required to understand the impact of 3q29del across the lifespan. The data analyzed in the present study were collected via the online 3q29 registry (3q29deletion.org); thus, internet access and basic technological capabilities are required for an individual to participate and we were unable to measure biological features that may correlate with the observed behavioral challenges. Future studies will be required to determine if biological features, such as structural or functional brain abnormalities, may contribute to the behavioral challenges documented in the present study. Finally, we note the possibility that the parents and caregivers motivated to participate in the online 3q29 registry may have more severely affected children than the population of individuals with 3q29del at large. However, the similarities between the data in the present study and prior deep phenotyping by our team (Klaiman, 2022; Murphy et al., 2018; Sanchez Russo et al., 2021), specifically relating to neurodevelopmental and psychiatric comorbidity, suggests that our sample in the present study is representative, and that neurodevelopmental and psychiatric comorbidity is a feature of 3q29del.

The results of this study add to our growing understanding of the behavioral phenotype of 3q29del. We show that an entirely web-based participant recruitment and data collection strategy to study a rare disorder such as 3q29del is an eminently feasible and effective approach, which is highly relevant in the post-COVID-19 clinical research environment and to the study of rare disorders at large. We found that the CBCL and ABCL can be effectively deployed as screening tools, even in populations such as individuals with 3q29del where a high degree of neurodevelopmental and psychiatric comorbidity can result in complex symptom presentation. This study builds on prior work by our team to identify neurodevelopmental and psychiatric comorbidity as a central behavioral feature associated with the 3q29 deletion; our evolving understanding of the phenotypic spectrum of 3q29del allows for improved treatment strategies that will maximize patient outcomes and long-term success.

## Supplementary Information

Below is the link to the electronic supplementary material.

Supplementary file1 (DOCX 1271 KB)

## Data Availability

The datasets used and/or analyzed during the current study are available from the Corresponding author on reasonable request.
