# Supplementary material for: Behavioral Phenotypes and Comorbidity in 3q29 Deletion Syndrome: Results from the 3q29 Registry
Source: J Autism Dev Disord. 2024 Jan 12;55(2):667–77. doi: 10.1007/s10803-023-06218-w (PMC11814048; doi:10.1007/s10803-023-06218-w)
Supplement: Supplementary file 1 — Supplementary file1 (DOCX 1271 KB) [file 10803_2023_6218_MOESM1_ESM.docx]

**Table S1.** Score cutoffs for CBCL and ABCL scales. Note that for the Competence and Adaptive Functioning scales the scoring is reversed relative to the Composite and DSM-keyed scales, such that lower scores on the Competence and Adaptive Functioning scales correspond to worse performance.

| **Scale Type** | **Normal cutoff** | **Borderline cutoff** | **Clinical cutoff** |
| --- | --- | --- | --- |
| Composite | <60 | 60-63 | >63 |
| DSM-keyed | <65 | 65-69 | >69 |
| School age CBCL Total Competence | >40 | 37-40 | <37 |
| School age CBCL Competence subscales | >35 | 31-35 | <31 |
| ABCL Adaptive Functioning | >35 | 31-35 | <31 |
|  | **Delayed** | **Normal** |  |
| Preschool CBCL Vocabulary Development | <=20 | >20 |  |
| Preschool CBCL Phrase Development | <=15 | >15 |  |

**Figure S1.** Venn diagram showing the distribution of scales across the preschool CBCL, school age CBCL, and ABCL.

AD/H, Attention-Deficit/Hyperactivity

**Figure 2.** **A)** Receiver operating characteristic curve showing the ability of the preschool CBCL DSM autism spectrum problems scale to correctly classify study participants with 3q29del based on parent-reported ASD diagnosis (n=32). **B)** Receiver operating characteristic curve showing the ability of the CBCL/ABCL DSM anxiety problems scale to correctly classify study participants with 3q29del based on previously published anxiety diagnosis (n=29) [1]. **C)** Receiver operating characteristic curve showing the ability of the CBCL/ABCL DSM AD/H problems scale to correctly classify study participants with 3q29del based on previously published ADHD diagnosis (n=29) [1].

**Figure S3. A)** Number of study participants with 3q29del and controls (n=6 3q29del, 6 control) scoring in the Normal and Delayed categories on the preschool CBCL Phrase Development scale. **B)** Number of study participants with 3q29del and controls (n=8 3q29del, 8 control) scoring in the Normal and Delayed categories on the preschool CBCL Vocabulary Development scale. **C)** Distribution of scores on the school age CBCL Competence section scales for study participants with 3q29del and controls (n=56 3q29del, 35 control). **D)** Distribution of scores on the ABCL Adaptive section Friends scale for study participants with 3q29del and controls (n=8 3q29del, 4 control).

*, p<0.05; ***, p<0.001

**Figure S4.** Sex-stratified distribution of scores on the **A)** shared CBCL/ABCL composite and DSM-keyed scales (n=58 3q29del male, 38 3q29del female), **B)** DSM-keyed scales unique to the preschool CBCL (n=19 3q29del male, 13 3q29del female), **C)** DSM-keyed scales unique to the school age CBCL (n=35 3q29del male, 21 3q29del female), and **D)** DSM-keyed scales unique to the ABCL (n=4 3q29del male, 4 3q29del female) for study participants with 3q29del. Dashed red lines denote the cutoffs for the Borderline and Clinical score classifications.

N, Normal; B, Borderline; C, Clinical; AD/H, Attention-Deficit/Hyperactivity

References

1. Sanchez Russo, R., et al., *Deep phenotyping in 3q29 deletion syndrome: recommendations for clinical care.* Genet Med, 2021. **23**(5): p. 872-880.
